# Hepatic angiomyolipoma: A case report and literature review

**DOI:** 10.1016/j.ijscr.2020.11.045

**Published:** 2020-11-11

**Authors:** Simone A. Günster, Mia Kim, Johan F. Lock, Katica Krajinovic

**Affiliations:** a Surgical Department 1, Clinical Center Fürth, Jakob-Henle-Straße 1, D-90766, Fürth, Germany; b Department of General, Visceral, Transplantation, Vascular and Pediatric Surgery, University Hospital of Würzburg, Würzburg, Germany

**Keywords:** Case report, HAML, Hepatic angiomyolipoma, Mesenchymal liver tumor, Hepatic lobectomy, Liver tumour

## Abstract

**Introduction:**

Hepatic angiomyolipoma (HAML) is a rare mesenchymal liver tumour which belongs to the family of perivascular epithelioid cell tumours (PEComas). It is typically composed of blood vessels, smooth muscle, and adipose cells, and shows strong immunoreactivity for HMB-45.

**Presentation of the case:**

A 57-year-old woman was referred to our hospital with an unclear liver lesion. A fine needle biopsy revealed a suspicion of hepatic angiomyolipoma with extramedullary haematopoiesis. Preoperative imaging revealed a tumour 17 cm in diameter in the left liver lobe segments II and III. A lobectomy of the left lobe segments II and III was performed. The pathological diagnosis of hepatic angiomyolipoma was obtained.

**Discussion:**

Variations in the predominance of the tissue components in HAML impedes diagnosis based on imaging alone. The most promising evidence of HAML is the histological identification of lipomatous, myomatous, and angiomatous tissue combined with immunohistochemical positivity for HMB-45. Although the tumour is considered benign, some cases have been described with malignant behaviour. Surgical resection should be considered in case of symptoms, inconclusive biopsy, or growth in follow-up. Other surgical indications may include aggressive patterns such as vascular invasion, p53 immunoreactivity, or rapidly proliferating tumour cells.

**Conclusion:**

HAML is a rare liver tumour. In patients with symptoms, uncertain diagnosis, or tumour growth, surgical resection should be performed according to oncological criteria.

## Highlights

• HAML is a rare mesenchymal liver tumour which belongs to the family of perivascular epithelioid cell tumours.
• HAML is typically composed of blood vessels, smooth muscle, and adipose cells.
• HAML is characteristically positive for HMB-45.
• In patients with symptoms, uncertain diagnosis, or tumour growth, surgical resection should be performed.

## 1 Introduction

Hepatic angiomyolipoma (HAML) is a rare, usually benign mesenchymal liver tumour that was first reported by Ishak et al. in 1976 [1]. It belongs to a group of perivascular epithelioid cell tumours called PEComa and is typically composed of blood vessels, smooth muscle, and adipose cells [[2], [3], [4]]. While the exact prevalence of HAML is unknown, estimations range between 300 and 600 cases worldwide [[4], [5], [6], [7], [8]].

We describe the case of a 54-year-old female patient with symptomatic HAML treated by left-sided lobectomy following the SCARE guidelines [9].

## 2 Presentation of the case

A 57-year-old woman was referred to our hospital with an unclear liver lesion. The tumour was found incidentally during the diagnostic clarification of thoracic pain by computed tomography (CT). The patient described abdominal discomfort and a feeling of fullness. She had no history of viral hepatitis, alcohol abuse, or any liver disease. In the clinical examination, the abdomen presented supple, without palpable resistance. However, deep palpation could cause mild tenderness in the epigastric region. Laboratory tests were normal including being negative for hepatitis B virus surface antigen and anti-hepatitis C virus antibody. Serum alpha-fetoprotein (AFP), carcinoembryonic antigen (CEA), and carbohydrate antigen 19-1 (CA 19-9) levels were all within normal limits. Drug and family history were unremarkable.

**Fig. 1:**
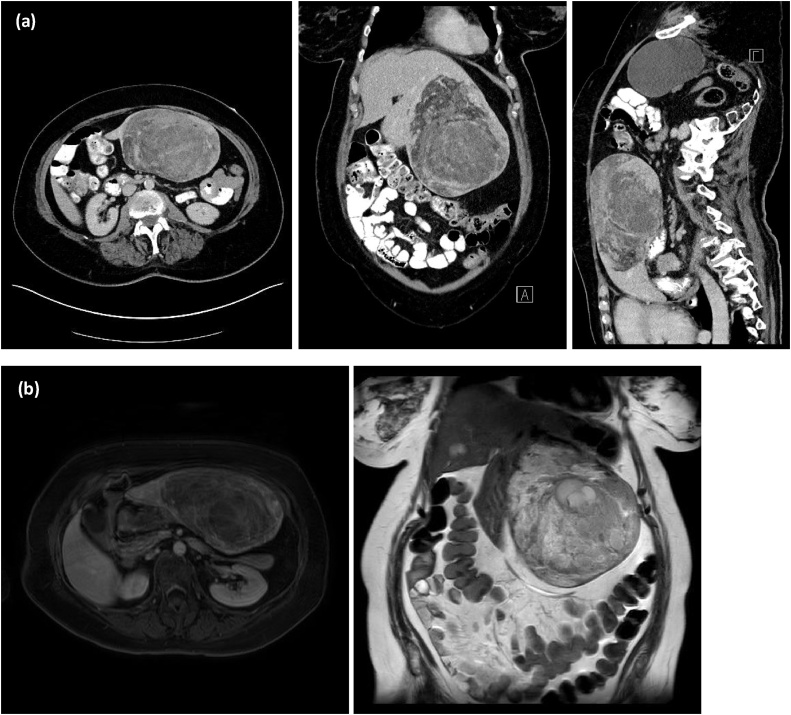
(a) Contrast-enhanced CT showed a 17 cm in diameter tumour in the left lobe of the liver. (b) MRI showed a 1250 mL volume mass with inhomogeneous intensity in T1- and T2-weighted sequences in left liver segments II and III with cystic and haemorrhagic components, a marginal hyperperfusion and diffusion restriction, and large fatty areas.

Contrast-enhanced CT (CECT) revealed a tumour 17 cm in diameter in the left lobe of the liver (Fig. 1(a)). Subsequent magnet resonance imaging (MRI) showed a 1250 mL volume mass in the left liver segments II and III with inhomogeneous intensity in T1- and T2-weighted sequences. In addition, there were cystic and haemorrhagic parts, a marginal hyperperfusion and diffusion restriction, and large fatty areas (Fig. 1(b)).

As imaging was inconclusive, a fine needle biopsy was indicated. Histologically, a mesenchymal neoplasia with lipomatous tissue and smooth muscle cells with extramedullary haematopoiesis and strong positivity for HMB-45 were found. The complementary colonoscopy and gastroscopy showed no pathological findings. In conclusion, surgical resection for suspected symptomatic hepatic angiomyolipoma with displacing growth was indicated.

**Fig. 2:**
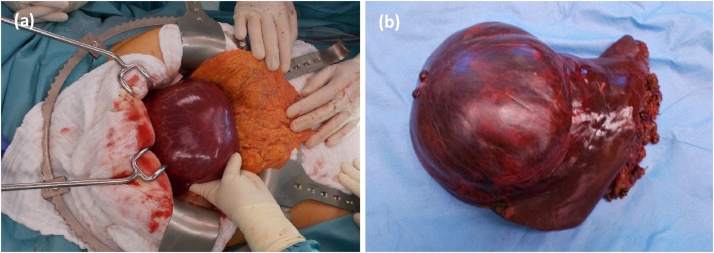
(a) Intraoperative view on the cystic tumour of the left liver. (b) Surgical specimen: Lobectomy on the left side of liver segments II and III with a cystic tumour measuring approximately 15 cm in diameter.

We performed a lobectomy on left liver lobe segments II and III. Intraoperatively, a cystic tumour measuring approximately 15 cm in diameter with a clear impression of the anterior stomach wall was found. Intraoperative ultrasonography of the liver was performed to define the limits for resection. The tumour was limited to liver segments II and III. Ultrasound did not show any infiltrative growth, so that the resection was performed as a left-sided lobectomy (Fig. 2).

Pathological examination of the resected tissue confirmed the initial diagnosis of HAML and showed a tumour measuring 16 × 15 × 6.5 cm with a lipomatous component of adipocytes and a myogenic component with partly spindle and partly epithelioid cell morphology. The myogenic component showed small foci of extramedullary haematopoiesis. Furthermore, a smaller, vascular component could be delimited in the tumour tissue. No atypia, mitoses or necrosis were detected. Immunohistochemistry consistently showed strong positivity for HMB-45.

The postoperative course was uneventful and the patient was discharged on the eighth postoperative day.

## 3 Discussion

Angiomyolipomas (AML) typically arise in kidneys. 50% of renal AML are associated with tuberous sclerosis, which is an autosomal-dominant inherited phacomatosis that is associated with mental retardation, epilepsy, adenoma sebaceum, and AML. The prevalence of HAML is reported to be 5%–15% in patients with tuberous sclerosis [3].

Although the tumour is considered benign, some cases with malignant behaviour such as invasive growth, metastasis, and recurrence after resection have been described [6,7,[10], [11], [12], [13]]. In a systematic review performed in 2017, the cumulative incidence of malignant behaviour of HAML is given as 4.1% [4]. Possible signs of malignancy mentioned in the literature are significant increase in size over a short period, a change in tumour composition, metastasis in other organs, recurrence after curative surgery or invasive growth into the vessels [14].

Previous studies have shown that HAML is usually found incidentally in asymptomatic patients [[15], [16], [17]] as the symptoms are mostly unspecific like abdominal discomfort or a feeling of fullness [18]. In terms of the differential diagnosis, almost all benign and malignant tumours of the liver are to be considered. Variations in the predominance of the tissue components impedes reaching a well-founded diagnosis based on imaging alone. Due to the lipomatous tissue, HAML frequently presents with hyperintensity in T1- and T2-weighted MRI sequences, as a heterogeneously hyperechoic mass on ultrasound, and as a homogeneous or heterogeneous low-density lesion in plain CT [14,19,20]. Abdominal angiography and biopsy can support the diagnosis when imaging is inconclusive. Immunohistochemically, *HAML* are characteristically positive for HMB-45, an antibody that responds to an antigen present in melanocytic tumours such as malignant melanoma [3,18,21,22]. Klompenhouwer et al. reported HMB-45 positivity in 91.5% of patients with HAML. Furthermore, in their systematic review from 2017, imaging provided the correct diagnosis for only 28.2% of the 292 patients with HAML, whereas biopsy and detection of HMB-45 led to correct diagnosis in 78.1% [4]. We conclude, therefore, that histological identification of lipomatous, myomatous, and angiomatous tissue with immunohistochemical positivity for HMB-45 provides the most reliable evidence of HAML.

Conservative management involving close follow-up is suggested for asymptomatic patients with histologically proven HAML smaller than 5 cm [6]. With a risk estimate of 0.8% in surgically treated patients, HAML-related mortality is very low. Surgical resection should be considered in patients with symptoms, inconclusive biopsy, or growth in follow-up according to oncological criteria [4]. Other possible surgical indications are aggressive patterns such as vascular invasion, p53 immunoreactivity, or rapid proliferation of the tumour cells [4,23]. Surgical resection can be performed open or laparoscopically according to oncological criteria [24].

## 4 Conclusion

HAML is a rare, usually benign, mesenchymal tumour of the liver that is often found incidentally in asymptomatic patients. If a reliable diagnosis of HAML can be made (by CT and MRI plus immunoreactivity for HMB-45), the tumour is less than 5 cm in size, and the patient has no symptoms, a conservative therapeutic regimen with close follow-up can be chosen. In case of symptoms, uncertain diagnosis, or tumour growth, surgical resection should be performed according to oncological criteria.

## Declaration of Competing Interest

The authors report no declarations of interest.

## Funding

No sources of funding.

## Ethical approval

Not applicable.

## Consent

Written informed consent was obtained from the patient for publication of this case report and accompanying images. A copy of the written consent is available for review by the Editor-in-Chief of this journal on request.

## Author contribution

Simone Andrea Günster wrote the manuscript.

Katica Krajinovic., Mia Kim and Johan Friso Lock reviewed/edited the manuscript and contributed to the discussion.

## Registration of research studies

N/A.

## Guarantor

Katica Krajinovic

Surgical Department 1, Clinical Center Fürth, Jakob-Henle-Straße 1, D-90766 Fürth, Germany.

Email: katica.krajinovic@klinikum-fuerth.de

Simone Andrea Günster

Surgical Department 1, Clinical Center Fürth, Jakob-Henle-Straße 1, D-90766 Fürth, Germany.

Email: simone-andrea.guenster@klinikum-fuerth.de

## Provenance and peer review

Not commissioned, externally peer-reviewed.
